# Human Tick-Borne Encephalitis and Characterization of Virus from Biting Tick

**DOI:** 10.3201/eid2208.151962

**Published:** 2016-08

**Authors:** Anna J. Henningsson, Richard Lindqvist, Peter Norberg, Pontus Lindblom, Anette Roth, Pia Forsberg, Tomas Bergström, Anna K. Överby, Per-Eric Lindgren

**Affiliations:** Region Jönköping County, Jönköping, Sweden (A.J. Henningsson, P.-E. Lindgren);; Umeå University, Umeå, Sweden (R. Lindqvist, A.K. Överby);; University of Gothenburg, Gothenburg, Sweden (P. Norberg, A. Roth, T. Bergström);; Linköping University, Linköping, Sweden (P. Lindblom, P. Forsberg, P.-E. Lindgren);; Linköping University Hospital, Linköping (P. Forsberg)

**Keywords:** tick-borne encephalitis, flavivirus, tick-borne encephalitis virus, case report, sequence analysis, RNA, cell culture, vector-borne infections, viruses

## Abstract

We report a case of human tick-borne encephalitis (TBE) in which the TBE virus was isolated from the biting tick. Viral growth and sequence were characterized and compared with those of a reference strain. Virus isolation from ticks from patients with TBE may offer a new approach for studies of epidemiology and pathogenicity.

Tick-borne encephalitis (TBE), a notable public health problem in many parts of Europe and Asia (*1*), is a severe infection affecting the central nervous system that may cause death or long-term illness (*2*). Most available sequences of the European TBE virus (TBEV) are derived from ticks, because the virus is rarely isolated from patients, and clinical samples generally are PCR negative at onset of neurologic symptoms (*3*–*5*). This situation hampers TBEV molecular studies. Furthermore, data are limited on viral transmission from ticks to humans. We describe a human case of TBE in Sweden and isolation and characterization of the TBEV isolated from the biting tick.

## Case Report

**Figure 1:**
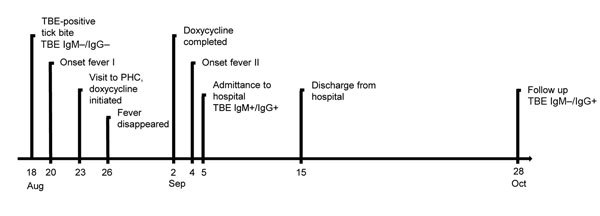
Time course of tick-borne encephalitis (TBE) in a 67-year-old man in Sweden, 2011. A classic biphasic onset of symptoms is shown. PHC, primary healthcare center.

On August 18, 2011, a 67-year-old man in Habo, Jönköping County, Sweden, noticed a tick bite on his foot (Figure 1). When he visited his primary healthcare center the same day, he was included a study of tickborne diseases (*6*,*7*); ethical approval was given by the Regional Ethics Committee, Linköping, Sweden, M132-06. He donated the tick and a blood sample and filled out a questionnaire. On August 20, 2011, the patient experienced fever, neck pain, and myalgia. On August 23, he again visited his primary health center with a temperature of 38°C. Lyme borreliosis was suspected, and doxycycline (200 mg/d for 10 d) was prescribed. After 3 days, the fever disappeared.

On September 4, the fever returned and the patient felt dizzy and experienced leg weakness. The following day, he returned to his primary health center with a temperature of 39°C, difficulty walking, and a severe headache. He was referred to the hospital, where a lumbar puncture was performed; cerebrospinal fluid (CSF) analysis revealed pleocytosis.

The patient was hospitalized for 10 days. He had difficulty walking, a headache, and pronounced fatigue but remained mentally lucid. Results of neurologic examination were normal in all other respects. Test results revealed high levels of serum TBEV IgM and IgG. He had not been vaccinated for TBE or yellow fever. CSF analyses for bacterial culture, *Borrelia burgdorferi* antibodies, varicella zoster virus DNA, and herpes simplex virus DNA were negative. The patient was discharged from the hospital on September 14 because his condition had improved. At a follow-up visit on October 28, he felt completely recovered, and a neuropsychiatric test showed no cognitive sequelae.

For study purposes, we obtained serum/plasma samples from the patient at inclusion in the tickborne disease study on August 18 and serum/plasma and CSF samples at hospital admission on September 5. We also obtained serum/plasma samples 2.5 months later. All samples were stored at −80°C. We analyzed TBEV antibodies in serum and CSF (IMMUNOZYM FSME [TBE] IgG and IgM; PROGEN Biotechnik GmbH, Heidelberg, Germany). In the serum samples from August 18, we could not detect any TBEV antibodies. The serum obtained on September 5 had high levels of TBEV IgG (368 Vienna units (VIEU)/mL) and IgM (571 VIEU/mL). No TBEV antibodies were detected in CSF. In the serum collected on October 28, the IgG titer had increased (>650 VIEU/mL), but IgM was not detectable.

TBEV quantitative PCR (qPCR) was performed as described (*7*) on all serum/plasma and CSF samples from the patient. TBEV RNA was not detectable in patient samples; however, no sample was obtained during the patient’s first fever episode, the assumed viremic phase.

We bisected the tick (*Ixodes ricinus* nymph) obtained from the patient using a sterile scalpel and extracted RNA from one half of it. TBEV was quantified by using qPCR (*7*), and high levels of TBEV RNA (>10^7^ copies) were detected. This result prompted us to isolate the new TBEV strain, tick/SWE/Habo/2011/1, directly from the tick, characterize its growth properties in cell culture, and sequence its genome.

The other half of the tick was homogenized in inoculation media (Dulbecco modified Eagle medium, 10X penicillin/streptomycin, 2% fetal calf serum, and 20 mM HEPES buffer) by FastPrep-24 (MP Biomedicals, Santa Ana, CA, USA) using lysing matrix A. After the mixture was centrifuged, we used the supernatant to inoculate VeroB4 African green monkey kidney cells. At 1 hour postinfection, cells were washed 3 times with inoculation media before infection media were added (Dulbecco modified Eagle medium, 1X penicillin/streptomycin, 2% fetal calf serum, and 20 mM HEPES buffer). The TBEV was harvested when cells showed cytopathic effects. Virus titers were determined by using a focus-forming assay (*8*).

**Figure 2:**
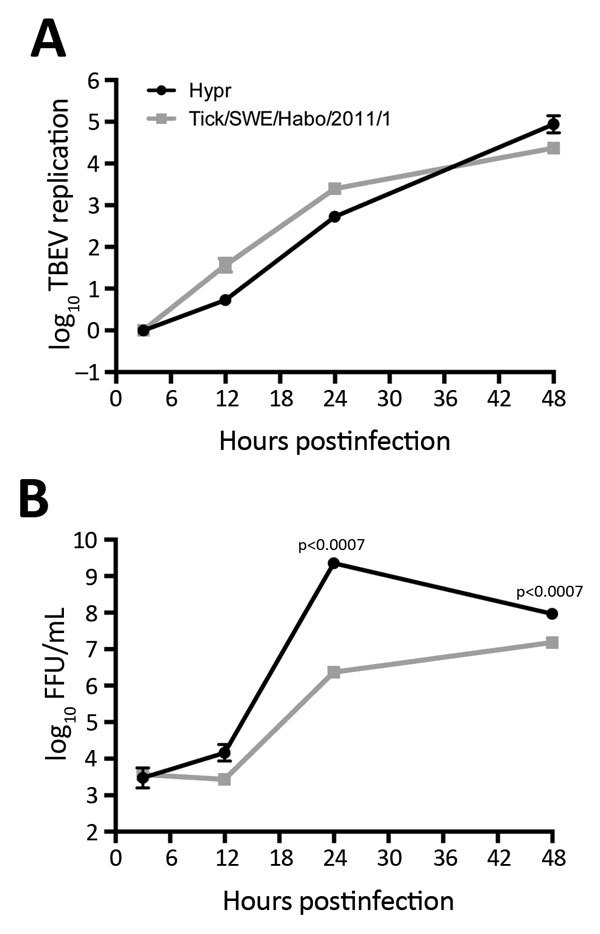
Time course of tick-borne encephalitis virus (TBEV) multiplication from sample from a 67-year-old man in Sweden, 2011. A549 cells were infected with the virus isolated from the tick in this study, tick/SWE/Habo/2011/1, and reference strain Hypr at multiplicity of infection 0.1. Total cellular RNA and cell culture supernatants were collected at different time points postinfection. A) Intracellular levels of viral RNA quantified by real-time reverse transcription PCR analysis. B) Virus titers in cell culture supernatants as determined by focus-forming assay (FFU). Mean values from 3 independent experiments are shown; error bars indicate SD. p values determined by Student *t* test.

A549 cells were infected with tick/SWE/Habo/2011/1 or Hypr 71 (*9*) at multiplicity of infection 0.1. Total RNA was extracted at different time points, and virus titers were determined with a focus-forming assay. Viral RNA was detected as previously described (*10*,*11*) (Figure 2). Tick/SWE/Habo/2011/1 replication was similar to that of the cell culture-adapted, highly pathogenic reference strain Hypr, originally isolated in Czechoslovakia from human blood (*9*). Progeny particle levels, however, were very different, indicating tick/SWE/Habo/2011/1 was less virulent.

We performed nucleotide sequencing of tick/SWE/Habo/2011/1 using 2 assays. First, deep sequencing was performed on the virus isolate. RNA was extracted from viral stock and real-time reverse transcription PCR was performed (*12*). The libraries were prepared by using modified TruSeq RNA Library Preparation Kit version 2 (Illumina, Inc., San Diego, CA, USA) and sequenced using MiSeq Desktop Sequencer (Illumina). De novo assembly was performed with Velvet algorithms (*13*), and a sequence of 10,899 nt was obtained. In the second assay, the strain was sequenced directly from PCR amplicons of RNA extracted from the tick (*14*) and yielded a sequence of 3,382 nt (nt 38–3,419). Sequences were submitted to GenBank under accession no. KU923573. 

When we compared the 2 sequences from these assays, we found homologous sequences even though 2 approaches and techniques had been used and the possibility that isolation in cell culture might constitute a selection. Results demonstrated a difference of 9 nt, all insertion/deletion mutations, between the deep-sequenced virus isolate from cell culture and the PCR-derived sequence obtained directly from the tick. Because any of these differences would lead to frame shifts in the genome, and such changes could not be detected in reference sequences of European TBEV strains, the insertion/deletion mutations most likely were artifacts. When we removed all gaps in the sequence alignment, the sequences were identical over the 3,382 nt. In addition to a comparison of 2 sequence methods, this result suggests that virus isolation did not introduce a selection bias in regard to the compared nucleotide sequences.

## Conclusions

Data on molecular epidemiology and TBEV transmission from ticks to humans are limited. This study shows that tick bites can cause TBE, confirming earlier epidemiologic associations. The median time between the tick bite and onset of the initial symptoms has previously been reported to be 8 days (range 4–28 days) (*15*). This case shows a shorter incubation period of 2 days, possibly because of a high viral load in the tick or because the TBEV strain was highly virulent. However, when growth characteristics were compared with those of the highly pathogenic Hypr strain, Hypr produced 1.000-fold more virus at 24 hours postinfection. The patient recovered without sequelae, which also indicates moderate virulence of the identified strain, tick/SWE/Habo/2011/1. TBE diagnosis was serologically confirmed, but unfortunately, TBEV could not be isolated from the patient samples, which has also been the case in previous studies (*4*,*5*).

Our findings demonstrate a strong link between a TBEV-infected tick and a patient with TBE, with an incubation time of only 2 days. PCR amplification, virus isolation, and genetic sequencing of TBEV from ticks detached from persons in whom TBE later develops may be a novel tool for studies of epidemiology and pathogenicity of this virus, which is difficult to isolate from patient samples.
